# Half-Yearly Report of Cases in Midwifery, Which Have Occurred in the Northern District of the London and Southwark Midwifery Institution

**Published:** 1830-02

**Authors:** C. Waller

**Affiliations:** Consulting Accoucheur to the above Institution, and Lecturer on Midwifery at the Medical School, 58, Aldersgate street.


### MIDWIFERY.

*Half-yearly Report of Cases in Midwifery, which have occurred in the Northern District of the London and Southwark Midwifery Institution.* By C. Waller, Esq. Consulting Accoucheur to the above Institution, and Lecturer on Midwifery at the Medical School, 58, Aldersgate street.

| 1829. | No. Women delivered. | Sex of Children: Males. | Females. | Born alive. | Stillborn. | Presentation. |
|---|---|---|---|---|---|---|
| July ...... | 21 | 11 | 10 | 20 | 1 | 20 Natural<br>1 Foot |
| August.... | 34 | 16 | 18 | 30 | 4 | 30 Natural<br>2 Breech<br>1 Funis & H.<br>1 Face to Pu. |
| September | 26 | 11 | 15 | 25 | 1 | Natural |
| October .. | 33 | 18 | 15 | 32 | 1 | Natural |
| November | 37 | 22 | 15 | 35 | 2 | Natural |
| December.. | 26 | 9 | 17 | 23 | 2 | 25 Natural<br>1 Breech |
| Total .... | 177 | 87 | 90 | 165 | 11 | |

During the first four months of this report, no case of interest occurred in the northern district of our Institution, with the exception of a fatal case of convulsions, in the eighth month of gestation. I was one morning requested to see this female, who was forty-one years of age, and was now pregnant for the first time. I found her with a feeble pulse, and an unhealthy florid-red appearance of the countenance. Her legs and thighs were enormously distended, from anasarcous effusion; there was great irritability of stomach, and, when the vomiting was urgent, she had occasional bleedings from the nose and mouth; her bowels were much constipated.

To relieve her legs, I made several punctures with a common sewing needle; and, with the hope of allaying the vomiting, and also of acting upon the bowels, I prescribed small doses of the sulphate of magnesia, giving directions to my pupil, Mr. Smith, to take a small quantity of blood from the arm, if the symptoms continued.

On the evening of the following day, I received an urgent message to visit her immediately, my informant stating that the patient was in fits; and on my arrival, I was told that the woman had been dead about a quarter of an hour.

At the commencement of the attack, a neighbouring surgeon was sent for, who had bled her in both arms, but without producing any mitigation of the symptoms.

With a small pocket bistoury I cut open the abdomen, and removed the child, but life was extinct.

The body was examined the next morning, the head with great care. From the sudden termination of the case, sanguineous effusion was anticipated: this opinion, however, was not correct; the blood-vessels were slightly turgid, and there was a generally œdematous state of the brain; in the ventricles there was also a small collection of fluid, and about three or four drachms (the quantity not measured) at the base of the brain. The abdominal viscera were perfectly healthy, the os uteri completely closed.

Two cases of what is absurdly called scirrhous adhesion of the placenta have occurred, one of them attended with a most alarming hemorrhage. This poor woman neglected to send for assistance till the child was nearly born, and on my pupil, Mr. Blair, arriving at her habitation, the infant was lying on the bed, and the blood pouring forth in immense quantities. Mr. B. very promptly applied cold and friction, and the discharge immediately ceased. As the placenta did not come away, my assistance was requested. The patient was lying in the state just described; the pulse very feeble, in fact, scarcely to be felt, but still not inordinately accelerated; the body generally was cool, but there was not that icy coldness which is so often met with in severe hemorrhagy. On examining the uterus externally, it was felt contracted to a certain degree, though of large size. As she had experienced pains, and the placenta had not been expelled, I introduced my hand, and with great difficulty succeeded in removing it: the attachment was so firm, that some little time was occupied in its separation.

Although the blood ceased to flow, the patient's condition was not improved; there was a distressing degree of faintness, although there was not complete deliquium; the pulse at times perceptible, at others not. Brandy, ammonia, and milk, were given her, with temporary advantage; but the patient soon relapsed. This state remained for upwards of four hours, when permanent improvement took place.

The propriety of performing transfusion was discussed, and my own opinion strongly expressed in its favor: not that what may be termed the mortal symptoms were actually present: there was not the deep and laborious inspiration; the cerebral functions were not disturbed; the patient remained tolerably quiet, being nearly free from jactitation, and her stomach for the first two or three hours retained the stimulus and the nourishment introduced into it. Still, hour after hour, no perceptible improvement took place in this female's condition, and, from the debilitated state she was in previous to her confinement, and from the immense quantity of blood she had lost, I felt confident that, although she might rally from the *immediate* effects of the hemorrhage, her ultimate recovery was rendered extremely doubtful, and I have more than once declared it to be my firm conviction, as sufficient trials have now been made to prove that the operation of transfusion is not necessarily dangerous, that we are called upon to perform it in cases of this kind, without waiting till the resources of our patient are drained to the last drop. Let it not, however, be supposed that I would recommend transfusion indiscriminately in all severe cases of hemorrhage: I am aware that in a very large proportion even of severe hemorrhages, it is by no means necessary; but I wish to caution the juniors of the profession against deferring it too long.

During the first few days after her confinement, this female appeared to be doing pretty well, with the exception of suffering from severe headach, and at the expiration of a week she was enabled, though with some difficulty, to sit up for a short time: she, however, felt extremely debilitated, had no lacteal secretion, and her bowels were occasionally very irritable. Her strength declined, and, from her lying in bed, the skin about the hips gave way, and considerable sloughs were formed, which added greatly to her sufferings, and she is now (four weeks after her confinement) lying, I fear, in nearly a hopeless state. Would this effect have been prevented if the operation of transfusion had been performed?

In two instances the child descended with the face opposed to the symphysis pubis, and the occiput to the sacrum: in one, the pains were very severe, and the child, after a few hours, was born without assistance; in the other, there were no strong bearing-down efforts, which rendered it eventually necessary to deliver with the forceps: this was accomplished by my friend and colleague, Mr. DOUBLEDAY, (in my temporary absence from town,) and both mother and infant went on favorably.

Several cases of abdominal inflammation have occurred, some peritoneal, others uterine: they were, however, attended with considerable power, and therefore the usual remedies, viz. bleeding, with the internal administration of calomel and opium, were in every instance successful.

Two cases of retained placenta have been reported; in both of which its detachment and expulsion appeared to have been effected by the exhibition of two of my usual doses of the secale cornutum (twenty-five grains of the powder) in warm tea. I was present at one of them; the other was under the superintendence of my industrious and intelligent pupil, Mr. SMITH.

93, *Bartholomew Close; January* 1830.

---

